# Anti-Obesity Attributes; UHPLC-QTOF-MS/MS-Based Metabolite Profiling and Molecular Docking Insights of *Taraxacum officinale*

**DOI:** 10.3390/molecules25214935

**Published:** 2020-10-26

**Authors:** Zain Ul Aabideen, Muhammad Waseem Mumtaz, Muhammad Tayyab Akhtar, Hamid Mukhtar, Syed Ali Raza, Tooba Touqeer, Nazamid Saari

**Affiliations:** 1 Department of Chemistry, University of Gujrat, Gujrat 50700, Pakistan; chzain321@yahoo.com (Z.U.A.); tuba.toqir@gmail.com (T.T.); 2 Institute of Industrial Biotechnology, GC University Lahore, Lahore 54000, Pakistan; tayyabakhtar@hotmail.com (M.T.A.); hamidmukhtar@gcu.edu.pk (H.M.); 3 Department of Chemistry, GC University Lahore, Lahore 54000, Pakistan; chemstone@yahoo.com; 4 Faculty of Food Science and Technology, University Putra Malaysia, Serdang 43400, Selangor, Malaysia

**Keywords:** *Taraxacum officinale*, antioxidant, obesity, metabolite profiling, molecular docking, lipid metabolism, obese mice

## Abstract

The naturopathic treatment of obesity is a matter of keen interest to develop efficient natural pharmacological routes for disease management with low or negligible toxicity and side effects. For this purpose, optimized ultrasonicated hydroethanolic extracts of *Taraxacum officinale* were evaluated for antiobesity attributes. The 2,2-diphenyl-1-picrylhydrazyl method was adopted to evaluate antioxidant potential. Porcine pancreatic lipase inhibitory assay was conducted to assess the in vitro antiobesity property. Ultra-high performance chromatography equipped with a mass spectrometer was utilized to profile the secondary metabolites in the most potent extract. The 60% ethanolic extract exhibited highest extract yield (25.05 ± 0.07%), total phenolic contents (123.42 ± 0.007 mg GAE/g DE), total flavonoid contents (55.81 ± 0.004 RE/g DE), DPPH-radical-scavenging activity (IC_50_ = 81.05 ± 0.96 µg/mL) and pancreatic lipase inhibitory properties (IC_50_ = 146.49 ± 4.24 µg/mL). The targeted metabolite fingerprinting highlighted the presence of high-value secondary metabolites. Molecular-binding energies computed by docking tool revealed the possible contribution towards pancreatic lipase inhibitory properties of secondary metabolites including myricetin, isomangiferin, icariside B4, kaempferol and luteolin derivatives when compared to the standard drug orlistat. In vivo investigations revealed a positive impact on the lipid profile and obesity biomarkers of obese mice. The study presents *Taraxacum officinale* as a potent source of functional bioactive ingredients to impart new insights into the existing pool of knowledge of naturopathic approaches towards obesity management.

## 1. Introduction

The prevalence of obesity continues to rise among all age groups and populations throughout the world. Obesity is a complex disorder that increases the risk of health impairments such as diabetes, metabolic syndrome, heart disease, cancer, stroke, etc. The mortality rate due to obesity is very high; it is the fifth-leading cause of death worldwide [1,2,3].

Pancreatic lipase, secreted from the pancreas, is an important enzyme responsible for the digestion of 70% of fats into monoglycerides and free fatty acids. Inhibition of this enzyme helps to reduce fat accumulation in adipose tissues [4]. Alternatively, lipoprotein lipase is an enzyme that catalyzes hydrolysis of triglycerides rich lipoproteins, very low density lipoproteins and chylomicrons resulting in release of nonesterified fatty acids and monoacylglycerol. An imbalance in lipoprotein lipase activity can affect the distribution of triglycerides between adipose tissues and muscles to cause obesity [5]. Therefore, the substances that can reduce the activity of lipases are considered to act as antiobesity agents. Escalation in oxidative stress can cause oxidative damage, which plays a leading role in the pathogenesis of many disorders such as cancer, obesity, diabetes, neurodegenerative diseases and atherosclerosis. Any action that can decrease oxidative stress would be therapeutically valuable. It is imperative to correlate the antioxidant and antiobesity properties of medicinal plants that are generally used in local medicinal systems to counter oxidative stress [6]. Prevention and treatment of obesity is crucial for healthcare systems, whose aim is to decrease the prevalence of obesity, overweight and related complications [7]. Obesity can be treated in number of different ways by physicians and other health care professionals including, diet regimes, exercise and pharmacotherapy. Currently used synthetic antiobesity drugs such as sibutramine and orlistat can be categorized into two major classes. Orlistat is synthetic inhibitor of gastrointestinal lipase that restricts the hydrolysis of triglycerides to reduce the subsequent absorption of monoglycerides and free fatty acids in intestine [8].

However, sibutramine is an appetite suppressant and works by means of low dietary intake [9]. However, antiobesity drugs are reported to cause severe side complications including insomnia, headache, dry mouth, constipation, high blood pressure and heart attack [10,11]. Therefore, investigations into medicinal herbs or plants that possess antioxidant and antiobesity activity is becoming a subject of keen interest due to their natural origin, low cost and limited adverse effects [12,13,14].

*Taraxacum officinale* (*T. officinale*)—of family Asteraceae—is commonly known as the dandelion. It is valuable plant and versatile in its nature, as the whole plant can be used for both medicine and food. *T. officinale* is used in traditional medicinal system to cure hepatic disorders due to its antioxidant and anti-inflammatory properties. The *T. officinale* is reported to reduce lipid accumulation and liver inflammation which may be vital factors in improving the liver function [15]. It has been reported that *T. officinale* roots and leaves induced hypolipidemic impact in high cholesterol diet fed rabbits by improving plasma antioxidant concentration which ultimately lead to prevention of oxidative stress related atherosclerosis [16]. Another study compared the effect of leaf and root extracts of *T. officinale* on triglyceride accumulation in 3T3-L 1 adipicytes. The comparison showed that, leaf extract exhibited greater anti-lipogenic impact on adipocytes than root extract. The study suggested that *T. officinale* leaves and roots might be utilized as an option to treat obesity in naturopathic mode [17]. The ethnopharmacological use of *T. officinale* is well established and its pharmacological properties may be due to presence of sesquiterpenes, saponins, phenolic compounds and flavonoids, supporting the long history of dandelion as a folkloric medicine [18].

The functional foods/nutraceutical industry is one of the rapidly growing industries globally, with excessive attributes towards improving health and managing chronic ailments. According to recent estimates, the global functional foods market size in 2018 was US $161.49 billion, and is projected to rise to US $275.77 billion by 2025 [19]. The awareness of functional foods is increasing, and its demand is growing even in developing countries. However, in Pakistan the situation regarding functional food formulation is very poor and needs special consideration.

The present investigation was designed to evaluate antioxidant and pancreatic lipase inhibitory potential, along with identification of phyto-constituents of hydroethanolic leaf extracts of *T. officinale* to provide scientific evidence regarding its traditional use for obesity management. The findings may be of great significance to utilize *T. officinale* for obesity management and functional food development with medicinal potential.

## 2. Results

### 2.1. Extract Yield, Total Phenolic and Flavonoid Contents

Extraction is an important step in studies involving biologic potential of plants. Functional molecules are usually occurred in low concentration in plant extracts. The high extract yields may guarantee the relatively higher amounts of active compounds. The role of solvent polarity and extraction techniques is imperative for high extract yields. Extracts with high polyphenol concentrations reflect high antioxidant and related pharmacological activities [20,21]. Phenolics are vital plant bioactives with the ability to scavenge free radicals for improvement in physiological attributes especially under diseased or stressed conditions. The antioxidant activities of polyphenols are mainly due to site specific interaction of their structural moieties with reactive oxygen species (ROS) [22]. Flavonoids being important polyphenolic compounds also impart a dynamic contribution to maintain the β-cells normal functioning by encountering ROS and other free radicals [23]. The antioxidant role of polyphenols also help to maintain energy homeostasis to prevent living systems and biomolecules from the possible oxidative injury to cause various ailments [12,24]. Hence, it is imperative to quantify the phenolics and flavonoids to predict about possible antioxidant and antiobesity potential of a particular plant extract or fraction. The experimental outcomes of current work regarding extract yields, total phenolic and flavonoids from leaves of *T. officinale* are presented in Table 1. It was noted that maximum extract yield was recovered in case of 60% hydroethanolic extract being significantly higher among all extracts (*p* < 0.05). The extract yield substantially increased by increasing ethanol component of solvent system up to 60% and then a decline was observed for further increase in the ethanol concentration. The solvent polarity set by 60% ethanol along with freeze drying and ultrasonication might be an effective combination to improve the extract yields [21]. The highest concentration of total phenolic contents (TPC) and total flavonoid contents (TFC) was computed in 60% hydroethanolic extract (123.42 ± 0.007 mg GAE/g, 55.81 ± 0.004 mg RE/g) followed by 80% extract (70.46 ± 0.004 mg GAE/g, 34.92 ± 0.003 mg RE/g), pure ethanolic extract (69.42 ± 0.005 mg GAE/g, 33.97 ± 0.004 mg RE/g) and 40% hydroethanolic extract (33.53 ± 0.003 mg GAE/g, 21.09 ± 0.005 mg RE/g). Minimum amounts of TPC and TFC were detected in 20% hydroethanolic extract (18.53 ± 0.004 mg GAE/g, 12.82 ± 0.001 mg RE/g). The extract yields and polyphenol concentration increased significantly by increasing the alcoholic fraction of solvent system up to 60% and then decreased upon further increase in ethanol. Comparatively larger amounts of polyphenols from 60% hydroethanolic extract may be due to polarity of the solvent which creates compatibility with polar compounds. A statistical comparison produced a statistically insignificant difference of means for TPC and TFC values among 80% hydroethanolic and pure ethanolic extracts (*p* > 0.05). A direct relation was observed between extract yields and polyphenols which might appear in antioxidant and biological activities. The strong linkage between phytochemicals present in plant extracts and antioxidant activity is well reported and established phenomenon [20,25].

### 2.2. Antioxidant Activity and Pancreatic Lipase Inhibition Assay

The results of DPPH scavenging and pancreatic lipase inhibition are given in Table 2. The results indicated that 60% ethanolic extract exhibited lowest IC_50_ value for DPPH scavenging which differed slightly from IC_50_ value for 80% ethanolic extract. However, this difference of activity was statistically significant (*p* < 0.05). The antioxidant potential possessed by plant extracts was most probably due to presence of polyphenols which exerted their reducing impact on DPPH free radical.

The antioxidant activity of plants is a prior indication of their possible medicinal potential. Antioxidants defend the body against disorders including Alzheimer’s disease, cancer, atherosclerosis and obesity. A low dietary intake of antioxidants is problematic, creating oxidative stress-oriented health disorders. A workable approach to this problem is the development of foods enriched with effective antioxidants from safe source like plants. The food fortification with natural and safe antioxidants can display an essential role in scavenging the ROS to mitigate oxidative stress and related complications [26,27]. The findings of DPPH assay confirmed the antioxidant properties of *T. officinale* leaves. The leaf extracts of *T. officinale* having high phenolic and flavonoid contents exhibited stronger DPPH scavenging which showed that polyphenols were the major contributing factor towards antioxidant properties. Again, the role of solvent system was of imperious nature to extract high polyphenols from leaves of *T. officinale* and this impact was reflected in antioxidant potential of leaf extracts. However, it was observed that the antioxidant activity of plant extracts followed dose-dependent behavior. High extract amounts may have greater antioxidant potential probably due to high concentration of active ingredients. A recent study on *Conocarpus lancifolius* confirmed that 60% ethanol was the most appropriate solvent system to get maximum amounts of phenolics and flavonoids for maximum antioxidant output and antidiabetic properties [20].

The IC_50_ values of pancreatic lipase inhibition were compared, and it was noted that all extracts showed considerable inhibition of pancreatic lipase activity. However, the statistical analysis signified 60% ethanolic extract as most effective and potent source for pancreatic lipase inhibitors (*p* < 0.05). The porcine pancreatic lipase inhibition by extracts of *T. officinale* was moderately effective than findings of previous works [8,28]. It was evident from the findings of current research that there was a direct relationship between polyphenol contents, antioxidant activity and pancreatic lipase inhibitory properties of leaf extracts of *T. officinale*. Therefore, 60% ethanolic extract being most potent may be a rich source of some lipase inhibitory agents to mitigate or reduce the intensity of obesity.

The inhibition of pancreatic lipase routes to reduce the lipids digestion and resultant fatty acid absorption through intestine. This phenomenon can be improvised by incorporating natural products and ingredients in foods for antiobesity traits. The inhibition of pancreatic lipase shown by plant extracts was likely ought to presence of secondary metabolites. The structural features of secondary metabolites or phytochemicals have the ability to bind with the amino acid residues of proteins to restrict their functions [29]. The pancreatic lipase is a potential pharmacological target to control obesity and does not involved complex mechanism of operation. Therefore, pancreatic lipase can be inhibited through blocking of functional residues. The phytochemicals as chemical markers may have potential to induce conformational changes in enzyme structure to inhibit its activity. The role of phytochemicals is pivotal in lipase inhibition and the identification of compound is highly needed in this regard. A study reported the antiobesity role of *T. officinale* in high fat diet fed rats, however secondary metabolites were not investigated [30]. The nature and type of functional metabolites must be known to execute the plant extracts for drug development and food fortification.

### 2.3. Metabolite Fingerprinting

**Figure 1:**
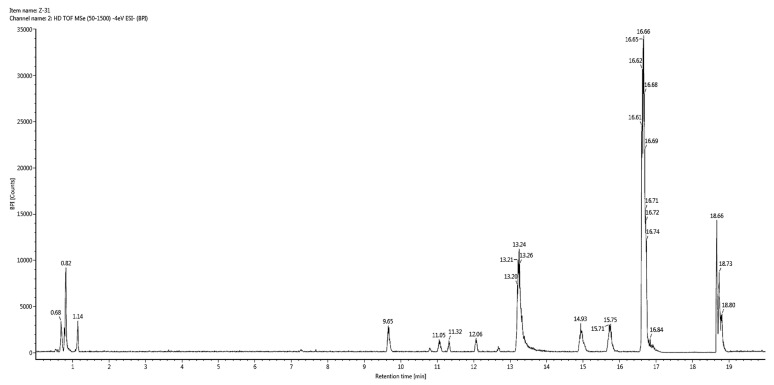
Base peak chromatogram for 60% hydroethanolic leaf extract from *T. officinale*.

**Figure 2:**
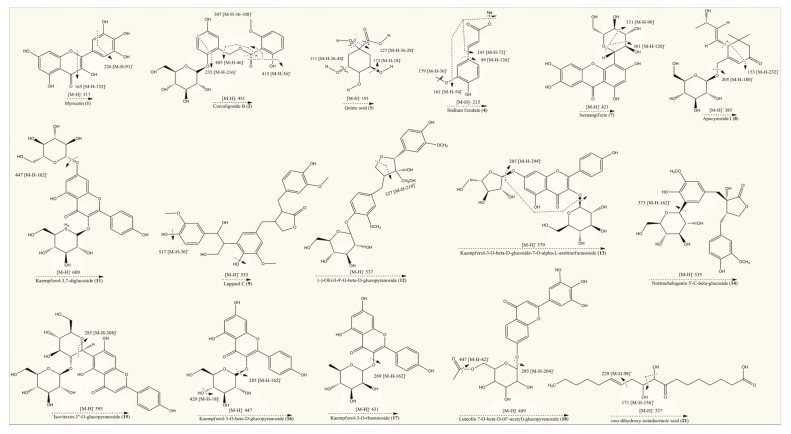
Fragmentation patterns of identified compounds in hydroethanolic extract of *T. officinale*.

In vitro studies revealed that 60% hydroethanolic extract from *T. officinale* leaf was most effective. Hence, the same was processed for identification of bioactive metabolites by UHPLC-QTOF-MS/MS. The base peak chromatogram of chromatographic analysis of said extract is given as Figure 1. Mass spectral data of all identified bioactives (including [M − H]^−^ ions, MS/MS fragments, retention times and class of compounds) is given in Table 3, while the fragmentation patterns of compounds are presented as Figure 2.

Precursor ions [M − H]^−^ were generated by negative ionization mode of mass spectrometer, which were further analyzed by product ion mode to produce their fragments or daughter ion peaks. Totally, 21 compounds peaks were analyzed for identification purpose. Compound (**1**) (retention time of 0.69 min) with deprotonated ion at *m*/*z* 317 and the molecular formula C_15_H_10_O_8_ was suggested as myricetin. The MS/MS fragment of the same compound was observed at 226, which indicated the possible loss of C_3_H_7_O_3_ (loss of 91). It also indicated characteristic fragment ion at 165 most probably due to the neutral loss of C_7_H_4_O_4_ (loss of 152) from the precursor ion [31]. Compound (**2**) (eluted at the retention time of 0.78 min) gave a deprotonated molecular [M − H]^−^ ion at *m*/*z* 451 (C_21_H_24_O_11_) [32]. The MS/MS spectrum of this compound presented fragments at 415, 405 and 235, indicating possible cleavage of 2H_2_O (loss of 36), C_2_H_6_O (loss of 46) and C_9_H_12_O_6_ (loss of 216) from the mother ion at 451, respectively while a characteristic fragment at 307 might be due to C_7_H_8_O (loss of 108) from the ion at 415. Based on the molecular composition and fragmentation mechanism, compound (**2**) was tentatively identified as curculigoside B. Compound (**3**) eluting at 0.79 min displayed precursor [M − H]^−^ ion at *m*/*z* 191. The same molecular ion was further fragmented into daughter ions at 173[M – H − H_2_O]^−^ (loss of 18), 127 [M – H − 2H_2_O − CO]^−^ (loss of 64), 111 [M – H − 2H_2_O − CO_2_]^−^ (loss of 80) and 85 [M – H − C_3_H_6_O_4_]^−^ (loss of 106), whereas the peak at 93 could be attributed to the phenol group. These fragmentations were consistent with previously reported studies [27,33]. Hence, compound (**3**) was confirmed as quinic acid on the basis of spectral information. Compound (**4**) was recorded at the retention time of 0.81 min in negative ionization mode and produced molecular ion peak at *m*/*z* 215 (C_10_H_9_NaO_4_). A prominent peak was also appeared at 179 (C_9_H_7_O_4_) after possible loss of CH_2_Na group (loss of 36) from parent ion. The deprotonated ion on further fragmentation led to the formation of fragment ions at 161 [M – H − CH_3_ONa]^−^ (loss of 54), 143 [M – H – C_3_H_4_O_2_]^−^ (loss of 72) and 89 [M – H – C_7_H_10_O_2_]^−^ (loss of 126).This fragmentation mechanism was consistent with the literature [33,34,35]. Based on QTOF-MS/MS data, characteristic fragment ions and reported literature, compounds (**4**) was most probably identified as sodium ferulate. Metabolite (**5**), with molecular formula C_4_H_6_O_5_ (recorded at retention time of 0.94 min) and having the precursor [M − H]^−^ ion at *m*/*z* 133 in the negative ionization mode, was characterized as 2-hydroxy-succinic acid and was confirmed by matching with literature data [36]. Compound (**6**) (eluting at 6.22 min) with parent ion peak at *m*/*z* 177 and the molecular formula C_9_H_6_O_4_ was identified as a daphnetin and agreed with the previously reported data [37]. The molecular ion of the compound (**7**) at *m*/*z* 421 [M − H]^−^ eluting at retention time of 7.14 min, produced the diagnostic fragment ion at 331, which was due to the cleavage of C_3_H_6_O_3_ molecule (loss of 90) from the ion at 421. The presence of daughter ion at 301 was due to loss of C_4_H_8_O_4_ (loss of 120) from the precursor ion. Based on spectral and literature data, compound (**7**) was tentatively assigned isomangiferin [38]. Compound (**8**) appeared at the retention time of 7.26 min and was identified as apocynoside I. The molecular ion at *m*/*z* 385 [M − H]^−^ (C_19_H_30_O_8_) and mass spectrum with product ions at 205 and 153 (which may be derived by cleavage of C_6_H_12_O_6_ (loss of 180) and C_10_H_16_O_6_ (loss of 232) residue from precursor ion, respectively) were in accordance with this compound [39]. Compound (**9**) was identified/characterized as lappaol C on the basis of its MS/MS spectrum and retention behavior, which was same as the standard lappaol C. The MS/MS spectrum of lappaol C indicated [M − H]^−^ ion of *m*/*z* 553 in negative ionization mode [40,41]. While the fragment at 517 may be due to the neutral loss of water molecules (loss of 36) from precursor ion. Compound (**10**) appeared at retention time of 7.78 min exhibited a mass spectrum with deprotonated [M − H]^−^ ion at *m*/*z* 387. Examination of mass data bank (PubChem) indicated these data to be consistent with the structure of icariside B4. Compound (**11**) eluting at the retention time of 7.96 min showed a molecular [M − H]^−^ ion at *m*/*z* 609 that generated a characteristic fragment ion at 447 ([M − H − 162]^−^, suggesting possible neutral loss of hexosyl residue). As per reported data, the fragment at 447 is typical for kaempferol glucoside [42]. Thus, compound (**10**) was identified tentatively as kaempferol-3,7-diglucoside. Metabolite (**12**) was identified as (−)-olivil-4′-*O*-β-d-glucopyranoside owing to precursor ions at *m*/*z* 537 [M − H]^–^ (C_26_H_34_O_12_) [43]. Still, MS/MS spectrum of compound (**12**) in negative ion mode indicated product ion at 327 may be due to neutral loss of C_10_H_10_O_5_ (5-hydroxyferulic acid group, loss of 210) from parent ion at 537. Compound (**13**) (retention time of 9.33 min, *m*/*z* 579 [M − H]^–^) exhibited a fragment at 285 [M − H − 294]^−^ (after possible cleavage of l-arabinofuranoside (loss of 132) and hexoside (loss of 162) residues). Furthermore, the fragment at 285 [kaempferol − H]^−^ was indicative of kaempferol derivative [44,45]. Compound (**13**) was therefore tentatively identified as kaempferol-3-*O*-β-d-glucoside-7-*O*-α-l-arabinofuranoside.

Compound (**14**) (eluting at retention time of 9.34 min) presented [M − H]^−^ ion at *m*/*z* 535 (C_26_H_32_O_12_) in negative ionization mode and its MS/MS spectrum exhibited product ion at 373, produced after possible loss of hexose moiety (loss of 162) from precursor ion. According to spectral information and the literature data [46], compound (**14**) was identified as nortrachelogenin 5′-*C*-β-glucoside. In negative ionization mode compound (**15**) gave the deprotonated molecular [M − H]^−^ ion at *m*/*z* 593, which corresponds to the molecular formula as C_27_H_30_O_15_ [47]. In MS/MS spectrum this compound showed a characteristic fragment at 285, may be due to successive removal of hexosyl (loss of 162) + rhamnosyl (loss of 146) moieties from parent ion. Therefore, metabolite (**15**) was tentatively identified as isovitexin-3″-*O*-glucopyranoside. Compound (**16**) (eluting at retention time = 9.66 min) exhibited deprotonated [M − H]^−^ ion at *m/z* 447. The MS/MS spectrum of this compound yielded the fragments at 429 may be due to neutral loss of water molecule. Moreover, the aglycone fragment at 285 (base peak) indicated the loss of a hexose moiety from precursor ion that generated a fragment ion at 256, consistent with kaempferol, as described previously [48,49]. Compound (**16**) was therefore identified as Kaempferol-3-*O*-β-d-glucopyranoside. Compound (**17**) recorded at retention time of 10.80 min, with a deprotonated [M − H]^−^ ion at *m/z* 431, was identified as kaempferol-3-*O*-rhamnoside according to MS/MS data and the data from reported literature. In mass spectrum of this compound the presence of a prominent fragment at 269 indicates the elimination of a glucosyl moiety (loss of 162) [50]. Compound (**18**), eluting at the retention time of 10.82 min was identified as luteolin 7-*O*-β-d-(6″-acetyl)-glucopyranoside. It produced a deprotonated ion at *m*/*z* 489 (C_23_H_22_O_12_) in negative ionization mode [51]. The precursor ion was further fragmented into product ion peaks at 447 and 285, produced by possible cleavage of C_2_H_2_O (loss of 42) and acetyl-hexose (loss of 204) units. Compound (**19**) (retention time of 11.32 min) was assigned as a derivative of heptanone. The tentative identification of this compound was carried out based on the comparison of its MS/MS data with that reported in previous literature [52]. Compound (**20**) (retention time of 15.72 min) with a deprotonated [M − H]^−^ ion at *m*/*z* 479, was identified as bruceine B. In MS/MS spectrum, this compound yielded three fragments at 429, 414 and 397, consistent with the findings of previous investigations [52,53]. Compound (**21**) presented a deprotonated molecular ion at *m*/*z* 327 and other major fragment ions at 229 and 171 (characteristic for an oxylipin compound), indicating possible removal of C_7_H_14_ (loss of 98) and C_9_H_16_O_2_ (loss of 156), respectively from precursor ion. Previously it is reported that oxo-dihydroxy-octadecenoic acid generated the daughter ion peaks at *m*/*z* 327, 291, 229, 211, 209, 171, 97 and 85. Based on reported literature, the compound was identified tentatively as oxo-dihydroxy-octadecenoic acid [54,55,56].

### 2.4. In Silico Molecular Docking

In silico docking analysis was conducted using molecular operating environment (MOE) to elucidate the binding profiles of phytochemicals identified in extract, with pancreatic lipase. The results were compared with that of orlistat (FDA approved standard drug for lipase inhibition). All compounds revealed good interaction with the amino acid residues present in active pocket of pancreatic lipase. Most of the compounds exhibited lower binding energy than orlistat which illustrated that they may inhibit pancreatic lipase activity to considerable extent. The binding affinities of all possible phytochemicals in extract and their interactions with amino acid residue of 1LPB are presented in Table 4. The bonding energies ranged from −8.6208 to −16.2939 kcal/mol. Isomangiferin and myricetin showed considerable binding energies, i.e., −16.2939 and −15.1097 kcal/mol, respectively.

**Figure 3:**
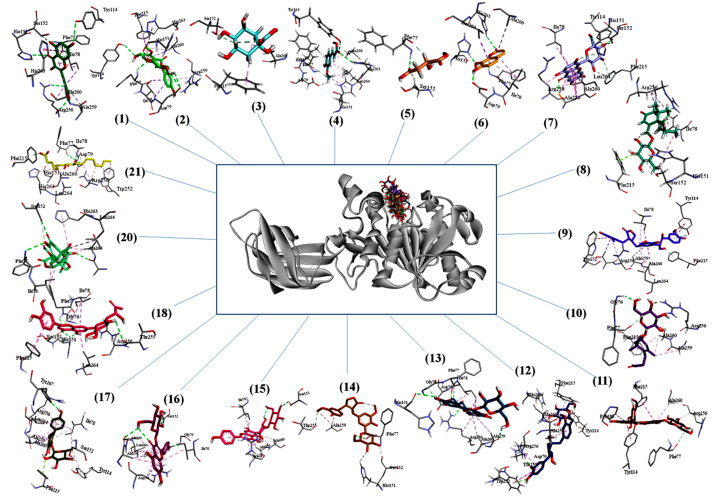
Three-dimensional binding poses of compounds on orlistat and interaction plots of phytochemicals with lipase enzyme.

The three dimensional binding pose of all compounds superimposed on orlistat and interaction plots between phytochemicals and enzyme are presented in Figure 3. Many significant interactions of docked phytochemicals with lipase were observed. Binding poses of most potent phytochemicals (based on binding energy) illustrate many similarities with the binding profile of orlistat. Orlistat shows hydrogen bonds with two key residues of active site, i.e., TYR114 and PHE77 and hydrophobic interactions with many other residues including most important amino acid SER152. Myricetin which have high binding affinity with lipase with binding energy of −15.1097, also revealed to have strong hydrogen bonds with TYR114, PHE77, however it has hydrogen bonds with three other amino acids HIS151, ALA260, ARG256 that makes more stable protein–ligand complex. Isomangiferin having highest binding energy was hydrogen bonded with TYR114, HIS151, SER152, ALA259, it was also able to interact with several hydrophobic interactions that included HIS151, ALA259, PHE215, ALA260, ILE78, ARG256, LEU264. The activity of pancreatic lipase (PDB No: 1LPB) is maintained by the catalytic triad (His263, Ser152 and Asp176 residues) present in active pocket of enzyme. Furthermore, Ser152 is the most important residue for lipolytic activity and any chemical modification of SER152 can demolish the activity. Therefore, a compound that can bind with triad, particularly with SER152 can inhibit lipolysis. Among phytochemicals identified in leaf extract, isomangiferin, kaempferol-3-*O*-β-d-glucopyranoside, isovitexin-3″-*O*-glucopyranoside and kaempferol-3-*O*-rhamnoside made strong hydrogen bonds with SER152. High binding affinities and interaction with key residues speculated that these plant based compounds can contribute significantly to pancreatic lipase inhibition activities.

The molecular docking analysis provided an insight of structural interaction playing deep inside the enzyme active site pockets. The findings elucidated the role of terminal hydroxyl groups and oxygen to develop H-bonding with amino acid residues of enzyme to produce energetically favorable conformations [57]. The numerous compounds like myricetin, isomangiferin, kaempferol derivatives and luteolin7-*O*-β-d-(6′’-acetyl)-glucopyranoside were detected in current study. The identified compounds were found to have favorable binding affinity even better than orlistat (positive control) and proved as excellent chemical markers to inhibit the activity of pancreatic lipase enzyme which may be a helping tool for drug development.

### 2.5. In Vivo Impact of Plant Extract on Lipid Profile and Blood Chemistry

**Figure 4:**
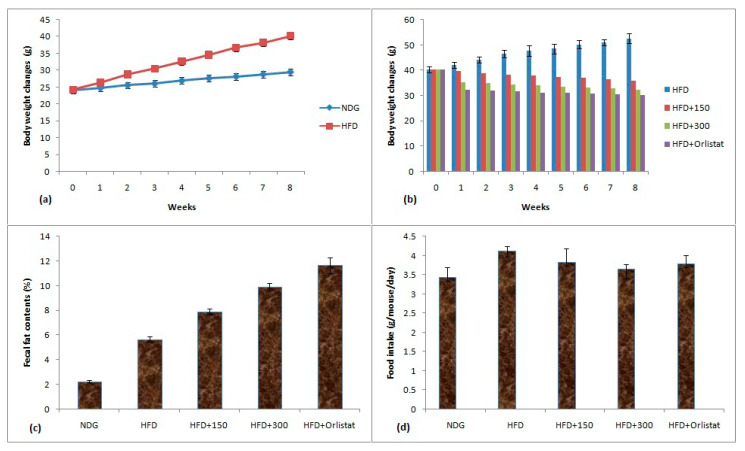
(**a**)Body weight changes of NDG and HFD mice; (**b**)body weight changes of mice treated with plant extract and orlistat; (**c**) fecal fat contents; (**d**) food intake by mice. Conditions: HFD group (having only HFD), HFD + 150 (having HFD + 150 mg/kg BW plant extract), HFD + 300 (having HFD + 300 mg/kg BW plant extract) and HFD + orlistat).

The 60% ethanolic plant extract being the most potent regarding antioxidant and pancreatic lipase inhibitory properties was selected for in vivo dosing. The comparison of weight gain of obese mice, their fecal fat contents and food intake is given in Figure 4a–d. The weight change data showed that an increase of 44.94% was observed for HFD mice whereas this increment for NDG was 22.21% at the end of eight weeks HFD treatment (Figure 4a). After eight weeks HFD treatment, the obese mice were split into groups to evaluate the influence of plant extracts on obesity parameters. The treatment with plant extracts and orlistat remained continue for further eight weeks period. The HFD mice treated with plant extract at 300 mg/kg BW restricted the weight to 32.22 ± 1.86 g when compared to HFD group (52.66 ± 2.03 g) at the end of eight weeks experimental protocol (Figure 4b). The orlistat also inhibited the weight gain and eight week’s value of BW of mice was 30.09 ± 1.61 g. The increase in body weights upon consuming HFD was most probably due to massive acylation of saturated fatty acids which were stored in adipose tissues. HFD also reported to reduce the satiety signal which resulted in weight gain [58].

The results of fecal fat contents are given in Figure 4c. The lowest fecal fat contents of 2.25% were observed for NDG mice while highest fat contents of 11.65% were noted for orlistat-treated mice. The high extract dose of 300 mg/kg BW substantially increased the fecal fat contents (9.92%) than HFD mice with fecal fat content value of 5.67%. The increase in fecal fat contents by plant extract was considered as an important phenomenon involved in antiobesity properties of plant extract [59]. The results of food intake are given as Figure 4d. The lowest food intake of 3.44 g/mouse/day was observed for NDG mice whereas highest food consumption of 4.12 g/mouse/day was noticed for HFD mice. The values of food intake for HFD + 300 and HFD + Orlistat groups were 3.65 and 3.8 g/mouse/day, respectively. There was a slight variation among food intake values during the period of eight weeks.

The mice were dissected and vital organs like liver, heart and kidney were collected, weighed and compared. The results of organ weight are given in Table 5. The HFD increased the liver, heart and kidney weights of mice. The comparison of liver weight showed that plant extract (HFD + 300) inhibited the increase in liver weight efficiently even higher than orlistat and statistical analysis revealed that the value of liver weight for HFD + 300 mice was significantly lower (*p* < 0.05). The heart and kidney weight were also lower in case of HFD + 300 mice, but not significantly lower than orlistat and NDG (*p* < 0.05). The restriction in organ weight gain upon administrating plant extract may be attributed to some metabolic changes, e.g., modifications in diets which activates the melanocyte hormone and promotes organ’s weight loss and reduction in triglycerides absorption resultant in organ and body weight loss. Moreover, reduction in total cholesterol and LDL concentration also leads to reduction in weight by regulating the adipogenic and lipogenic transcription factors [60,61].

The lipid profile analysis, hemoglobin (Hb), ALT and AST are given in Table 6. It was clear from the values that plant extract at 300 mg dose significantly improved the lipid profile of obese mice by modulating the TC, HDL and LDL values. The *T*. *officinale* extract at 300 mg dose improved the TC by attenuating the LDL level and this amelioration in TC was significantly different from HFD (*p* < 0.05). The same dose also improved the Hb level of obese mice. The levels of ALT and AST were also lowered in animals having plant extract dose of 300 mg and ALT concentration was comparable to the values observed for orlistat-treated group (*p* > 0.05).

HFD resulted in a significant increase in TC, HDL and LDL level of mice. Previous studies have shown that dietary intake of saturated fats and cholesterol is associated with an increase in HDL and LDL and a positive correlation exists between high fat diets and serum HDL [62,63].

Modification in lipid metabolism occurred in mice treated with plant extracts which was reflected in improved lipid profile. The currently observed lowering of TC was most probably due to phytochemicals present in extract. The phyto-constituents being an important entity of plants, executed their ability to increase lipid metabolism in liver to reduce body weight [64]. The improvement of lipid profile of mice upon administrating plant extract was most probably due to synergistic effect and antioxidant potential of secondary metabolites [20]. The phytochemical profiling of 60% ethanolic extract of *T. officinale* indicated that most compounds were flavonoids in nature. Flavonoids are well known for their hypolipidemic impacts in living system. Flavonoids were reported to reduce TC and LDL levels by activating β-adrenergic receptors to burn fats and also inhibit adipogenesis by inducing apoptosis in preadipocytes of mice [65].

The HFD fed mice showed significantly higher AST and ALT levels than ND fed mice. Comparatively, higher levels of AST and ALT of HFD mice were most probably due to obesity generated metabolic abnormality in liver of obese mice [66]. Role of genetic responses could not be ignored in improvement of adipogenic situation during obesity development and propagation. The gene expression analysis was reported to play an important role to improve the blood biochemistry including lipid profile in adipogenesis. A study indicated that mRNA expression levels of PPAR-γ and C/EBPα were significantly downregulated which may be responsible for reduction in AST and ALT blood concentration [67]. The variation in AST and ALT concentrations were also of enormous consideration in living system suffering from obesity. The AST and ALT were reported to be transferred to plasma due to obesity operated oxidative damage to tissues [68]. Another study reported that AST and ALT were toxicity indicators and phytochemicals present in various plants reduced their plasma concentrations by improving liver and kidney functions [69]. The antioxidant role of phytochemicals is of pivotal nature which not only reduced obesity by improvising multiple site oriented metabolic processes, but also normalized the organ’s function. The phytochemicals especially polyphenols are well reported to improve the antioxidant defense line of body to mitigate obesity and related inflammations [70]. The myricetin, a flavonoid detected in 60% ethanolic extract of *T. officinale* leaf was reported to have antiobesity and hypolipidemic activities. Metabolite profiling of *T. officinale* extract revealed the presence of many kaempferol derivatives. Kaempferol and its derivatives are well known biologically functional molecules of plants. Kaempferol glycosides purified from Jindai soybean were reported to exhibit antiobesity properties by downregulation of PPAR-γ and SREBP-1c in high fat diet fed mice. Similarly, daphnetin was also reported to reduce lipid accumulation by upregulating the PI3K expression. Daphnetin was also responsible for lowering of reactive oxygen species in hepatocytes. The phytochemicals identified in 60% ethanolic extract probably exerted their synergistic impact to exert the antiobesity attributes of *T. officinale* [71,72,73].

## 3. Materials and Methods

### 3.1. Reagents and Chemicals

Folin–Ciocâlteu (FC) reagent, 2,2-diphenyl-1-picrylhydrazyl (DPPH), butylatedhydroxyanisole (BHA), gallic acid, rutin, NaNO_2_, NaOH, AlCl_3_, Tris-HCl buffer, pancreatic lipase, Arabic gum, acetone, olive oil, CH_3_OH, free fatty acids (FFA) used were analytical research grade (Sigma-Aldrich, BDH and Merck, Taufkirchen, Germany).

### 3.2. Extraction Optimization

Mature fresh *T. officinale* leaves were acquired from periphery of Azad Jammu & Kashmir, Pakistan. The plants were collected and retained carefully for identification. The identification of plant species was conducted at Botany Department of University of Gujrat, Pakistan and voucher specimen (UOGCHEM47/2018) was also submitted. Freshly collected leaves were cleaned gently to remove dust. The cleaned leaves were immediately treated with liquid N_2_ to stop the metabolic processes for maximum conservation of secondary metabolites. They were then subjected to lyophilization at −68 °C for 48 h to obtain fully dried fluffy material. The freeze dried leafy material was converted into fine powdered (60mesh) and stored in Ziplock packing at −80 °C till further experimentation. The powder (10 g) was then extracted in 100 mL of binary solvent system (ethanol: water solvent, i.e., 20:80, 40:60, 60:40, 80:20, *v/v* and pure ethanol) at ambient conditions of temperature (35 ± 0.2 °C) and humidity (25 ± 5%) for 2 days. After that obtained extracts were vortexed for 2 h (Wise Mix SHO1D, DAIHAN Scientific, Seoul, Korea) followed by sonication at 20 KHz for 30 min (Soniprep 150 ultrasonicator MSE, Buckinghamshire, UK), centrifuged at the rate of 13,000 rpm for 10 min and filtered using filtration assembly attached with a vacuum pump for proper removal of debris. Evaporation of excess solvent was carried out using a rotary vacuum evaporator under reduced pressure to avoid loss to phyto-constituents. Finally, the extracts were subjected to another freeze drying at −68 °C and stored at very low temperature in freezer till future use.

### 3.3. Total Phenolic and Flavonoid Contents

A previously established method was used to determine total phenolic contents (TPC) of the tested extracts with slight modifications [74]. For this purpose, extracts were dissolved in methanol and 200 µL of this extract fraction was mixed with FC reagent. The obtained mixture was left to rest for 5 min time period. After 5 min stay, the contents were mixed with 4mL of 20% Na_2_CO_3_. After an incubation period of 90 min at ambient temperature, the samples were subjected to absorbance measurement at 750 nm wavelength. The result of TPC was expressed as milligrams of gallic acid equivalent per gram (mg GAE/g).

An already reported AlCl_3-_based method was used to quantify total flavonoid contents (TFC) of extracts [75]. The plant extracts were dissolved in methanol and 200 µL of this dilution was taken for sample preparation. A reaction mixture was prepared by mixing 0.5 M NaNO_2_ (0.10 mL), 0.3 M AlCl_3_·6H_2_O (0.15 mL) and 30% MeOH (3.4 mL) followed by addition of 200 µL of prepared methanolic extract. After 5 min stay at room temperature, 1 mL of 1M NaOH was mixed with the reaction mixture and absorbance was noted at 510 nm spectrophotometrically. TFC were quantified by utilizing a flavonoid rutin (standard) and findings were reported as milligrams of rutin equivalent per gram dried extracts (mg RE/g DE).

### 3.4. Antioxidant Activity

Spectrophotometric method involving DPPH scavenging by plant extracts was adopted for determination of antioxidant activity. This method is based upon the bleaching of violet color of DPPH reagent to yellow by polyphenols present in extracts [76]. The DPPH scavenging was an index of antioxidant potential and measured as IC_50_ (µg/mL). A standard antioxidant compound (BHA) was also used as reference standard. The plant extracts (50–250 µg) were added to freshly prepare methanolic DPPH reagent solution. After addition of plant extracts, the samples were stayed for a period of 20 min at 35 °C for reaction completion. After incubation, the samples were subjected to absorbance measurement at 517 nm. The results of radical scavenging % were computed by using following mathematical relation:

Scavenging effect (%) = (Control_absorbance_ − Sample_absorbance_/Control_absorbance_)

### 3.5. Pancreatic Lipase Inhibitory Assay

Pancreatic lipase inhibitory potential of extracts was determined to assess the in vitro antiobesity properties. Plant extracts were added to porcine pancreatic lipase (dissolved in 0.01 M of Tris-HCl buffer) followed by addition of olive oil mixed with gum Arabic. The gum Arabic was used to homogenize the mixture of olive oil with water contents of sample. A well-established method was adopted to determine the pancreatic lipase inhibition of plant extracts [77] with minute changes. Plant extracts were dissolved in methanol and 0.5 mL of this dilution was mixed with lipase reaction mixture. The resultant solution was incubated for 30 min at 4 °C for completion of reaction followed by addition of 2 mL of substrate. Resulting mixture was incubated at 37 °C for 30 min time period. The ethanol and acetone (1:1) were added to reaction mixture to stop the reaction. The 0.02 M NaOH was used as titrant to neutralize free fatty acids till the pH reached to 9.4. The % inhibition of enzyme activity was calculated.

%Inhibition = 100% − ((Vs/Vc) × 100)

The vs. and Vc were the volume of base used for sample and control, respectively.

### 3.6. Metabolite Fingerprinting

Based upon data of different assays, the most potent extract was dissolved in MeOH (aq.). The methanolic extract was filtered for sample preparation through Poly-tetrafluoroethylene filter (0.45 µm). The UHPLC-QTOF-MS/MS (Agilent, Santa Clara, CA, USA) technique was used for metabolite identification due to its robustness, rapidness, efficacy and authenticity. The instrument was operated with scanning range of 50 to 1200 *m*/*z* (negative ionization mode). The solvent system was composed of water and acetonitrile each having 0.1% formic acid. Gradient elution was performed, and flow of mobile phase was set at 0.8 mL/min with sample injection volume of 20 µL. Sciex Peak views 2.1 software (Sciex, Selangor, Malaysia), ACD/Lab and ChemSpider/PubChem databases were utilized for data analysis [27]. Resolved peaks of spectrum were further characterized by considering the fragmentation patterns of identified compounds from previously reported studies [50,51,52,53,54,55,56,57].

### 3.7. In Silico Molecular Docking

The identified secondary metabolites were subjected to docking studies. For the purpose, Molecular Operating Environment (MOE 2016.08) software (Chemical Computing Group, Köln, Germany) was utilized. The porcine pancreatic lipase structure was downloaded, and compounds were docked in the active site region. The ligand–enzyme conformations were prepared, and binding energies were computed after minimizing the energy. The hydrophobic and hydrophilic interactions at various amino acid residues were studies. The docking result analysis of surface with graphic representation was done using MOE and discovery studio visualize (3ds, Tokyo, Japan).

### 3.8. In Vivo Hypolipidemic Investigation and Blood Biochemistry

The antiobesity impact of plant extract was evaluated in high fat diet (HFD) fed BALB/c mice. The 30 male mice (6 weeks old) with average body weight (BW) of 24.20 ± 0.985 g were arranged from the animal house of GC University Lahore and acclimatized for a period of 10 days (Arrive guidelines 2.0). A prior approval for animal study was obtained from the ethical committee of GC University Lahore vide letter GCU/IIB/006 dated 1st January 2019. After this, mice were allowed ad libitum supply of HFD and water for an eight-week period to increase their body weight. Six mice were fed on normal chow diet rather than HFD for comparison. HFD was composed of 30% protein, 50% corn starch, 10% sucrose, 5% corn oil, 2.5% cholesterol, 4% vanaspati ghee, 1% coconut oil, 10% milk butter, 5% minerals and 1% vitamins. Whereas normal diet contained 30% protein, 50% corn starch, 10% sucrose, 5% corn oil, 5% minerals and 1% vitamins [20].

After completion of an eight-week period, the BW of mice were measured and obese mice were further categorized into HFD group (having only HFD), HFD + 150 (having HFD + 150 mg/kg BW plant extract), HFD + 300 (having HFD + 300 mg/kg BW plant extract) and HFD + 50 mg/kg BW orlistat). The carboxy methyl cellulose (CMC) was used as vehicle for plant extracts and orlistat.

The mice were treated as per design for a further eight-week period. After completion of treatment protocol, mice BW were compared, and fecal fat contents were determined. The dietary intake was also recorded. The blood from the lateral vein of the mice was subjected to determine the total cholesterol (TC) in mg/dL, high density lipoproteins (HDL) in mg/dL, low density lipoproteins (LDL) in mg/dL and triglycerides in mg/dL [20]. The blood hemoglobin was also determined in g/dL. The changes in liver function test enzymes (alanine aminotransferase (ALT) and aspartate aminotransferase (AST)) were calculated to observe the changes in biochemical profiles of mice. Mice were dissected by applying lignocaine and ether anesthesia. All the ethical guidelines were followed to reduce the suffering of animals from experimental procedure. Mice were dissected to collect liver, heart and kidney with care and subjected to weight determination.

### 3.9. Statistical Analyses

Triplicate experimental runs were made and standard deviation (±) was applied. The difference of means was calculated using ANOVA at significance level of 0.05 to assess significance difference of means using Minitab 17.0 statistical software (Minitab Ltd., Coventry, United Kingdom). The significantly different values did not share a letter in results.

## 4. Conclusions

This study confirms the hypolipidemic attributes of 60% *T. officinale* extract, which not only improved the lipid profile, but also lowered the AST and ALT concentration of obese mice. Sixty percent ethanol was the most appropriate choice to increase extract yields to a significant level. Sixty percent ethanolic extract also exhibited comparatively higher phenolic and flavonoid contents. The DPPH-radical-scavenging and pancreatic lipase inhibition was also determined in terms of IC_50_ value. Sixty percent ethanolic extract posed lowest IC_50_ value which reflected highest antioxidant property and pancreatic lipase inhibitory potential. The UHPLC-QTOF-MS/MS-based metabolite profiling confirmed the presence of numerous compounds of pharmacological significance belonging to diverse classes of phytochemicals. Molecular docking tool also confirmed the pancreatic lipase inhibitory action of identified secondary metabolites. The majority compounds showed stronger bindings with lipase enzyme than standard compound orlistat in terms of binding energies. The in vivo trials revealed that there was substantial improvement in lipid profile and blood biochemistry of obese mice treated with 300 mg/kg body weight dose of 60% *T. officinale* leaf extract. The plant extract also imparted its impact on body organ weights. The *T. officinale* can be employed as a potential candidate for the naturopathic approach to manage obesity and functional food development with enormous biological properties. The currently presented information may be extended regarding isolation of phytochemicals for their individual antiobesity properties.

## Figures and Tables

**Table 1:** Results of extract yields, total phenolic contents (TPC) and total flavonoid contents (TFC).

Extracts	Extract Yields (%)	TPC in mg GAE/g DE	TFC in mg RE/g DE
20% ethanolic	16.1 ± 0.09 ^e^	18.53 ± 0.004 ^d^	12.82 ± 0.001 ^d^
40% ethanolic	19.32 ± 0.12 ^d^	33.53 ± 0.003 ^c^	21.09 ± 0.005 ^c^
60% ethanolic	25.05 ± 0.07 ^a^	123.42 ± 0.007 ^a^	55.81 ± 0.004 ^a^
80% ethanolic	22.76 ± 0.06 ^b^	70.46 ± 0.004 ^b^	34.92 ± 0.003 ^b^
Pure ethanolic	22.10 ± 0.14 ^c^	69.42 ± 0.005 ^b^	33.97 ± 0.004 ^b^

Superscript ^(a–e)^ indicates significant difference of means (*p* < 0.05). Values not sharing a letter categorized as significantly different.

**Table 2:** DPPH scavenging and pancreatic lipase inhibitory activity of hydroethanolic extracts.

Solvent	DPPH Activity (IC_50_ = µg/mL)	Pancreatic Lipase Inhibition (IC_50_ = µg/mL)
20% ethanolic	117 ± 1.27 ^f^	179.89 ± 3.43 ^f^
40% ethanolic	98.98 ± 2.03 ^e^	165.09 ± 2.03 ^d^
60% ethanolic	81.05 ± 0.96 ^b^	146.49 ± 4.24 ^b^
80% ethanolic	85.85 ± 1.55 ^c^	159.18 ± 3.06 ^c^
Pure ethanolic	91.11 ± 1.03 ^d^	176.03 ± 3.01 ^e^
BHA	16.55 ± 1.11 ^a^	–
Orlistat	–	12.24 ± 0.12 ^a^

Superscript ^(a–f)^ indicates significant difference of means (*p* < 0.05). Values not sharing a letter categorized as significantly different.

**Table 3:** Mass spectral information of all compounds identified in hydroethanolic leaf extract of *T. officinale*.

Sr No.	Retention Time (min)	[M − H]^−^ (*m*/*z*)	Predicted Formula	MS/MS Fragments (*m*/*z*)	Identification	Classification
1	0.69	317	C_15_H_10_O_8_	226, 165	Myricetin	Flavonoid
2	0.78	451	C_21_H_24_O_11_	415, 405, 307, 235	Curculigoside B	Phenolic glucoside
3	0.79	191	C_7_H_12_O_6_	173, 127, 111, 93, 85	Quinic acid	Organic acid
4	0.81	215	C_10_H_9_Na_4_	179, 161, 119, 89	Sodium ferulate	Ferulic acid
5	0.94	133	C_4_H_6_O_5_	Not fragmented	2-Hydroxy-succinicacid	Organic acid
6	6.22	177	C_9_H_6_O_4_	Not fragmented	Daphnetin	Coumarin
7	7.14	421	C_19_H_18_O_11_	331, 301	Isomangiferin	Xanthone
8	7.26	385	C_19_H_30_O_8_	205, 153	Apocynoside1	Ionone glucoside
9	7.29	553	C_30_H_34_O_10_	517	Lappaol C	Lignan
10	7.78	387	C_19_H_32_O_8_	Not fragmented	IcarisideB4	Ionone glycoside
11	7.96	609	C_27_H_30_O_16_	447	Kempferol-3,7-diglucoside	Flavonoid
12	8.05	537	C_26_H_34_O_12_	327	(−)-Olivil-4′-*O*-β-d-glucopyranoside	Lignan
13	9.33	579	C_26_H_28_O_15_	285	Kempferol-3-*O*-β-d-glucoside-7-*O*-α-l-arabinofuranoside	Flavonoid
14	9.34	535	C_26_H_32_O_12_	373, 285	Nortrachelogenin5′-*C*-β-glucoside	Flavonoid
15	9.44	593	C_27_H_30_O_15_^−^	285	Isovitexin-3″-*O*-glucopyranoside	Flavonoid
16	9.66	447	C_21_H_20_O_11_	429, 285, 256	Kempferol-3-*O*-β-d-glucopyranoside	Flavonoid
17	10.80	431	C_21_H_20_O_10_	269	Kempferol-3-*O*-rhamnoside	Flavonoid
18	10.82	489	C_23_H_22_O_12_	447, 285	Luteolin7-*O*-β-d-(6″-acetyl)-glucopyranoside	Flavonoid
19	11.32	723	–	713, 677, 659	Derivative of heptanone	–
20	15.72	479	C_23_H_28_O_11_	429, 414, 397	Bruceine B	Quassinoid (lactone)
21	15.74	327	C_18_H_32_O_5_	229, 171	Oxo-dihydroxy-octadecenoic acid	Fatty acid

**Table 4:** Binding energy data of secondary metabolites docked in pancreatic lipase.

Sr No.	Compound Name	Binding Energy kcal/mol	Interactions with Amino Acid Residues of 1LPB	
			Hydrogen Bonding	Non Bonding
1	Myricetin	−15.1097	PHE77, HIS151, TYR114, ALA260, ARG256	SER152, HIS263, ILE78, ALA260, ALA259, ARG256
2	Curculigoside B	−12.6517	TYR114, PHE77, HIS151, ASP79	PHE77, PHE215, HIS263, ARG256, ILE78, SER152, GLY76, ALA260
3	Quinic acid	−11.3489		PHE215, ALA260, SER152
4	Sodium ferulate	−10.8178	TYR114, HIS263	ALA260, ILE78, PHE77, HIS151, ASP79, LEU264
5	2-Hydroxy-succinicacid	−11.1306	PHE77	PHE77, SER152
6	Daphnetin	−11.0778	PHE77, HIS263, HIS151, ASP79	SER152, ALA260, LEU264, ILE78
7	Isomangiferin	−16.2939	SER152, TYR114, HIS151, ALA259	HIS151, ALA259, PHE215, ALA260. ILE78, ARG256, LEU264
8	Apocynoside 1	−12.9674	ARG256	ARD256, 1LE78, HIS151, PHE215, SER152
9	Lappaol C	−10.8984		TYR114, PHE215, ARG256, TRP252, ALA259, ALA260, ILE78, ASP79, LEU264
10	Icariside B4	−12.2148	ARG256, GLY76	PHE77, ALA260, ALA259, PHE215
11	Kempferol-3,7-diglucoside	−12.3240		TYP114, PHE215, PHE77, ARG256, ALA260, PRO180
12	(−)-Olivil-4′-*O*-β-d-glucopyranoside	−11.0636	ARG256	ARG256, TRP252, PHE215, ALA260, ALA260, THR255, ASP79, HIS263, TYR114
13	Kempferol-3-*O*-β-d-glucoside-7-*O*-α-l-arabinofuranoside	−14.0556	HIS151, ALA259, ARG256, ASP79	ALA259, ARG256, PHE77, GLY76, ALA260, ILE78
14	Nortrachelogenin-5′-*C*-β-glucoside	−12.2669	PHE77	SER152, HIS151, THR255, ALA259
15	Isovitexin-3″-*O*-glucopyranoside	−12.9536	SER152	ARG256, ALE78, ALA260, ALA259
16	Kempferol-3-*O*-β-d-glucopyranoside	−12.8289	SER152, ALA259, GLY76	ALA259, LEU264, ALA260, 1LE78, ARG256
17	Kempferol-3-*O*-rhamnoside	−13.9530	SER152, TYR267	PHE215, TYR114, ALA259, ILE78, ARG256, ALA260, LEU264
18	Luteolin-7-*O*-β-d-(6″-acetyl)-glucopyranoside	−13.9305	PHE77, HIS151, THR255, ARG256	ARG256, PHE215, ILE78, LUE264, SER152, GLY76
20	Bruceine B	−8.6208	PHE77, HIS263	HIS263, SER152, ALA260, LEU264, ILE78
21	Oxo-dihydroxy-octadecenoic acid	−11.6910	PHE77, ASP79	TRP252, ARG256, ILE78, HIS263, HIS151, LEU264, ALA260, PHE215
22	Orlistat (Standard drug))	−9.1309	PHE77, TYR114	TYR114, SER152, PRO180, PHE215, ILE78, ALA260, ARG256, ALA259, LEU264

**Table 5:** Liver, heart and kidney weight changes of understudy mice.

Treatments	Organ Weight (g)		
	Liver	Heart	Kidney
NDG	1.85 ± 0.04 ^b,c^	1.12 ± 0.03 ^a^	1.45 ± 0.03 ^a^
HFD	2.24 ± 0.07 ^e^	1.68 ± 0.07 ^e^	1.95 ± 0.02 ^e^
HFD + 150	2.16 ± 0.04 ^d^	1.60 ± 0.04 ^d^	1.87 ± 0.05 ^d^
HFD + 300	1.78 ± 0.05 ^a^	1.33 ± 0.04 ^c^	1.58 ± 0.04 ^c^
HFD + Orlistat 50 mg/kg BW	1.81 ± 0.04 ^a,b^	1.29 ± 0.06 ^b^	1.48 ± 0.05 ^a,b^

Superscript ^(a–e)^ indicates significant difference of means (*p* < 0.05). Values not sharing a letter categorized as significantly different.

**Table 6:** Lipid profile and biomarkers of understudy mice.

Parameters	ND	HFD	HFD + 150	HFD + 300	HFD + Orlistat (50 mg/kg BW)
TC in mg/dL	46.55 ± 1.87 ^a^	154.12 ± 5.45 ^e^	112.07 ± 3.55 ^d^	72.87 ± 2.03 ^c^	54.05 ± 2.05 ^b^
HDL (mg/dL)	25.66 ± 2.38 ^a^	85.50 ± 2.12 ^e^	61.27 ± 2.09 ^d^	38.04 ± 1.38 ^c^	32.55 ± 1.25 ^b^
LDL (mg/dL)	18.77 ± 0.73 ^a^	65.50 ± 3.84 ^e^	48.17 ± 3.22 ^d^	32.33 ± 0.95 ^c^	19.82 ± 0.75 ^a,b^
Triglyceride (mg/dL)	1.79 ± 0.03 ^b^	2.91 ± 0.08 ^d^	2.03 ± 0.02 ^b,c^	1.97 ± 0.04 ^b,c^	1.47 ± 0.03 ^a^
Hb (g/dL)	9.92 ± 0.11 ^a^	7.35 ± 0.15 ^e^	7.82 ± 0.09 ^d^	8.44 ± 0.13 ^b,c^	8.48 ± 0.11 ^b^
ALT (U/L)	25.25 ± 2.25 ^a^	80.05 ± 2.50 ^e^	72.10 ± 2.48 ^d^	45.39 ± 1.90 ^b,c^	42.84 ± 2.24 ^b^
AST (U/L)	40.12 ± 2.38 ^a^	76.24 ± 2.35 ^e^	63.98 ± 3.32 ^d^	51.11 ± 3.33 ^c^	47.35 ± 2.06 ^b^

Superscript ^(a–e)^ indicates significant difference of means (*p* < 0.05). Values not sharing a letter categorized as significantly different.
